# Validation of the 24-h perceived exertion recall survey in women in rural Tigray, Ethiopia

**DOI:** 10.1016/j.cdnut.2023.100064

**Published:** 2023-03-08

**Authors:** Jenna Golan, John F. Hoddinott

**Affiliations:** Division of Nutritional Sciences, Cornell University, Ithaca, NY, United States

**Keywords:** physical exertion, malnutrition, Ethiopia, nutritional status

## Abstract

**Background:**

Physical activity affects nutritional status and health. Currently, there are few validated survey tools for estimating physical activity in rural areas of low-income countries, including Ethiopia, which limits the ability of researchers to assess how physical activity affects nutritional status.

**Objectives:**

This study used accelerometry to validate 2 in-person questionnaires, the global physical activity questionnaire (GPAQ) and the 24-h perceived exertion recall survey (PERS).

**Methods:**

This study recruited 180 women aged between 18 and 45 y living in rural Tigray, Ethiopia. Participants had previously participated in an impact evaluation of a public work safety net. They wore an accelerometer for 8 d and responded to perceived exertion questionnaires twice. Data were collected on 89 women during the short rainy period and 91 women during the main rainy season. A survey method was considered valid if the proportion of time spent in moderate or vigorous physical activity (MVPA) levels had a Pearson’s correlation coefficient of >0.40 to the proportion of time spent in MVPA recorded by accelerometry.

**Results:**

The GPAQ had high reliability, but the overall validity was poorer than accelerometry. The proportion of time spent in MVPA according to the accelerometer was associated with discordance between GPAQ and accelerometry. MVPA levels, as measured by the 24-h PERS, had a fair agreement with accelerometry. The agreement increased to moderate/acceptable when adjusted for season and BMI.

**Conclusions:**

The 24-h PERS is a valid tool for estimating the physical activity of women living in rural highland Ethiopia. It can be used in future research to understand the physical activity demands of living in rural highland Ethiopia, enabling more targeted programs to address undernutrition.

## Introduction

The rising triple burden of malnutrition—undernutrition, micronutrient deficiencies, and overweight/obesity—is an increasingly important area of public health concern in low- and middle-income countries. Two forms of malnutrition—undernutrition and overweight/obesity—are a consequence, in part, of energy imbalances. Prolonged negative energy balance results in undernutrition, which has significant health risks [1]. For example, undernutrition during pregnancy is associated with fetal growth restrictions, the cause of 12% of child deaths worldwide; stunting in childhood; obesity and noncommunicable diseases in adulthood; and developmental deficits that can negatively affect school performance and income-generating potential [[2], [3], [4], [5]]. There is a lack of well-designed studies on the impact of undernutrition on maternal outcomes [4,6], but some evidence shows an increased risk of maternal morbidity [7] In Ethiopia, the focus of this study, in 2016, 22% of adult women were underweight [8].

A positive energy balance contributes to overweight/obesity. Globally, an estimated 2.2 billion people are overweight, of whom 772 million, or 12.3% of adult men and 16.2% of adult women, are obese. In 2010, 9.2% of adult men and 13.2% of adult women were obese [9]. Overweight and obesity during pregnancy are associated with maternal morbidity, preterm birth, and increased infant mortality [2]. A study in France found that risk of maternal death increased by 65% in overweight women and was more than double in obese women [10]. In Ethiopia, the proportion of women who were overweight or obese increased from 3% in 2000 to 8% in 2016 [8]. In men, the proportion increased from 2% in 2011 [11] to 3% in 2016 [8].

In low- and middle-income countries, efforts to reduce malnutrition (such as the USAID-funded Feed the Future initiative and the Bill and Melinda Gates Fund’s Alive and Thrive program, both operate in Ethiopia) focus on increasing energy intake and improving dietary quality [12]. A program specific to Ethiopia is the Productive Safety Net Programme (PSNP). In the rural highland regions of Ethiopia, the PSNP offers beneficiaries food or cash payments for labor on public works projects [13]. The impacts of the PSNP and other nutrition-sensitive interventions on food security and dietary intake are increasingly well characterized [13,14], in part due to the ability of researchers to collect data on these indicators through survey tools such as 24-h dietary recalls.

Efforts to assess the causes of malnutrition in all its forms, and the effectiveness of interventions designed to attenuate the prevalence of malnutrition, have benefited from decades of research on how best to measure food intake. However, 1 factor that may contribute to both underweight and overweight/obesity, physical activity, has received far less study. One reason for this lack of data is that criterion methods to measure physical activity, such as indirect and direct calorimetry, are expensive and have limited applicability in resource-constrained settings [15,16]. Indirect calorimetry either uses bulky machinery, which disrupts the activities of free-living adults, or doubly labeled water, which requires access to a laboratory and trained technician [17,18]. Direct calorimetry is confined to a laboratory and cannot measure energy expenditure in free-living persons.

Technology advancements have resulted in additional objective methods to measure physical activity exertion levels [19]. Triaxial accelerometers record activity in free-living persons [15,20,21] using unobtrusive devices that do not hamper physical activity. However, they still require significant resources to collect and analyze data, making them less feasible for data collection in large populations.

Survey tools would provide significant insight into the physical activity between and within populations. However, the few survey tools that have been developed have not been validated in many low-income countries, including rural Ethiopia [22]. The WHO developed the global physical activity questionnaire (GPAQ) as the global standard for monitoring physical activity [23,24]. The GPAQ measures physical activity “in a typical week.” During a face-to-face interview, respondents report how many days in a typical week and how much time in a typical day is spent in either moderate or vigorous activity across 3 domains: work and home, travel to and from places, and recreational activities. In addition, respondents report how much time is spent sitting or reclining on a typical day in sedentary behavior. Researchers then calculate the amount of time per week spent in each level of exertion during a week [23].

The validity of GPAQ to measure physical activity has been assessed in the United Kingdom [25], Bangladesh [26], and New York City [27]. Previous attempts to validate it in Ethiopia used pedometers, not triaxial accelerometers [22]. Researchers concluded that the GPAQ is more accurate in European and Asian settings than in physically active populations in sub-Saharan Africa [22]. Subsequently, the GPAQ has been used to measure sedentary behavior in populations with high rates of obesity [[28], [29], [30]].

There have been few efforts to validate physical activity questionnaires that include more detail over shorter periods of time. Therefore, we developed a novel 24-h perceived exertion recall survey (PERS). This survey collects information on time use and perceived energy exertion from the previous day through face-to-face interviews. It was adapted from the American Time Use Survey [31] and the time allocation module of the Women’s Empowerment in Agriculture Index [32], which was validated and used in Ethiopia, India, Bangladesh, and Malawi [[33], [34], [35]].

The 24-h PERS collects information on the participants’ primary and secondary activities and their perceived energy exertion during 30-min epochs starting an hour before sunrise and concluding an hour after sunset. Participants list all the activities they participated in during the previous day, the activities are then placed in chronological order, and the participants estimate the amount of time spent in each activity. Participants report their perceived exertion as sedentary, light, moderate, or vigorous in intensity for each activity, with the option of varying their exertion level for each epoch. Self-reported exertion level is preferable to using the Compendium of Physical Activity [31,36] due to the nature of manual labor in resource-limited settings such as Ethiopia. Studies in Australia [37], the United States [38], and China [39] have used this approach.

Neither the GPAQ nor the 24-h PERS has been validated in rural Ethiopia. To address these gaps in the literature, we collected and analyzed accelerometry data to assess 2 questionnaire-based methods of assessing physical activity, testing the reliability and validity of the GPAQ, and validating and calibrating a novel 24-h PERS in women living in rural Ethiopia. We test the hypotheses that *1*) the GPAQ will have moderate/acceptable reliability, *2*) the GPAQ will have moderate/acceptable validity compared with accelerometry data, *3*) the 24-h PERS will have moderate/acceptable validity compared with accelerometry data, and *4*) the agreement of the 24-h PERS will increase when calibrated using participant characteristics.

## Methods

### Study setting

This study was based in the Central, Eastern, and South zones of the Tigray region of Ethiopia. Tigray is located in northern Ethiopia. It is landlocked, and the terrain is mountainous. The road network is limited; very few households own motorized vehicles or bicycles, so travel on foot is the primary mode of transportation. Few households are connected to an electrical grid or have piped water, so water and firewood must be collected frequently. Livelihoods are based on rain-fed agriculture. In the Southern Zone of Tigray, the rainy season occurs between June and October, with crops harvested between October and December [14]. Income-generating activities are limited during the rest of the year. At the time of this study, during the dry season between January and June, many households in Tigray participated in the government-run PSNP. The PSNP is a public works program; participants work on activities such as road rehabilitation, reforestation, and construction of water storage ponds in return for a daily wage paid in cash or in-kind, wheat, or maize.

### Participant selection

We sought to recruit 96 women participants. This sample size was selected based on precedents set by earlier studies that had 45 participants [28], 78 participants [29], and several hundred participants [15]. Selection criteria were participants’ households also participating in an ongoing quantitative study of the impact of the PSNP on the nutritional status of women and children; were women; and were aged between 18 and 45 y with a child aged 6–23 mo [40].

Data collection occurred in Tigray’s Central, Eastern, and South zones. Within each zone, 4 counties where the earlier impact evaluation occurred were randomly selected. Within each county, villages were screened to ensure that they contained ≥2 eligible households that received benefits from the public works component of the PSNP (beneficiaries) and 2 eligible households that received no benefits (nonbeneficiaries); villages that did not meet this criterion were excluded. Two eligible villages were randomly selected per county. Within the villages, 2 PSNP and 2 non-PSNP households were randomly selected. There were 4 participants per village, yielding a target sample of 96 participants (96 = 4 participants per village × 2 villages per county × 4 counties per zone × 3 zones).

Participants were excluded if they were pregnant or planning on leaving the area during data collection. When unable to recruit the intended number of beneficiaries due to pregnancy, travel, or participants declining to participate, additional nonbeneficiaries were recruited. Efforts were made to include the same women in both rounds of data collection. When not feasible, additional eligible participants were recruited to maintain the sample size.

### Physical activity measures

Reference data for validation were collected using ActiGraph wGT3X-BT accelerometers (ActiGraph Corp). The ActiGraph wGT3X-BT is a triaxial accelerometer that records movement in the transverse, frontal, and sagittal planes. They are a reliable measure of physical activity [41] and have been used in various settings, including validating the GPAQ in the United Kingdom [25], France [42], and India [43]. Participants were instructed to wear the accelerometers around their waist and to remove them only when bathing or swimming. They were encouraged to wear the devices while sleeping, but were told to remove them if uncomfortable. Heart rate monitors were not used in this study; they were too large and bulky for the study participants.

Accelerometers recorded data in 1-s epochs with sampling frequencies at 30 Hz [44]. Data were processed, cleaned, and analyzed using ActiLife 6 software (ActiGraph). The raw count data were assessed for wear and nonwear time using the Choi et al. [45] algorithm. Insufficient wear time was defined as <12 h of recorded wear time during a day; a valid week required ≥5 valid days. The raw data were reintegrated into 10-s epochs, and Crouter’s refined 2-regression model [41,[46], [47], [48], [49]] was used to calculate metabolic equivalents (METs). Validated cut points are then applied to categorize METs into different exertion levels [50,51].

The main physical activity outcome variables included the proportion of recorded time spent in sedentary/light exertion, moderate exertion, vigorous exertion, and moderate or vigorous exertion. The proportion of time was used rather than total minutes due to the potential difference in total recorded time between the GPAQ and the accelerometers. For consistency, the proportion of time was also used for the 24-h PERS analysis.

Two physical activity survey modules, the GPAQ and the PERS, were validated using accelerometry. The GPAQ was analyzed according to the guidelines set out by the WHO [23]. Briefly, the data were first checked for implausible values; for example, observations were excluded if a participant reported implausible values, such as spending >16 h/d or >7 d/wk in an activity. Next, the average time per week reported in the different levels of physical exertion, including moderate or vigorous physical activity (MVPA), was calculated for the individual domains and pooled. Specific to this analysis, the proportion of time spent in each level of exertion was calculated by dividing the estimated average weekly time by the total amount of time in a week.

The second survey module was a 24-h PERS. This survey collected information on time use and perceived energy exertion from the previous day through face-to-face interviews. Data were excluded from the PERS if participants reported implausible amounts of time spent in high levels of exertion, such as an entire day at vigorous exertion. Next, the time spent in each level of exertion was calculated for each 24-h recall period. The proportion of time spent at each level of exertion was calculated.

### Data collection

Data collection took place over 2 4-wk periods. The dry season round, April–May 2019, occurred when minimal agricultural activities took place, and the PSNP was operational. The agricultural round, September–October 2019, occurred during the main rainy season when households were engaged in farming activities, and the PSNP was nonoperational.

Participants wore the accelerometer around their waist for 8 successive days, which permitted 7 full days of data collection on physical activity. During data collection, participants were visited 6 times to ensure the accelerometers were correctly worn and had sufficient battery. Participants responded to the GPAQ and 24-h PERS on the second and sixth visits. During the second visit, participants were weighed in duplicate (Seca 874, Seca), and they provided information on their age, education, and whether their partner currently resided in the same household. Additional data were drawn from information collected as part of an ongoing impact evaluation of the impact of the PSNP on the nutritional status of women and children [40]. This included participant characteristics, including height, education, dietary diversity, and lactation status; household characteristics, including household members, sources of income, food security [40], and housing quality; and village characteristics, including distance to markets, cities, and water sources.

### Statistical analysis

Data were analyzed using Stata version 14.2 (StataCorp). The cutoffs Pearson’s correlation coefficient (ρ) and Spearman’s rank correlation coefficient (ρ) for both reliability and validity were as follows: a coefficient between 0 and 0.2 was poor agreement, 0.21 and 0.40 was fair agreement, 0.41 and 0.60 was moderate/acceptable agreement, 0.61 and 0.80 was substantial agreement, and 0.81 and 1.0 was near-perfect agreement. These cutoffs were used in a multicountry study to assess the reliability and validity of the GPAQ [22].

### GPAQ analysis

The reliability of the GPAQ was assessed by comparing the data collected during the second visit to the final visit. The κ statistic and percent agreement were used for dichotomous responses, for example, a participant spent time in an activity [52], and Spearman’s rank correlation coefficient for continuous responses, for example, the amount of time spent in an activity [53]. For the GPAQ to be reliable, the agreement for both dichotomous and continuous responses needed to be moderate/acceptable (ρ > 0.40) [22]. The reliability of the GPAQ was tested separately for each survey round and for the rounds pooled.

The validity of the GPAQ was determined by comparing the proportion of time participants reported spending at each level of exertion to the proportion of time recorded by accelerometry. The participants’ responses to the GPAQ were averaged by round. Values were compared using Pearson’s correlation coefficient and Spearman’s rank correlation coefficient. For the GPAQ to be valid, the correlation coefficient needed to be moderate/acceptable.

An additional analysis used linear regression prediction models to identify predictors of discordance between the GPAQ and accelerometer. Discordancy was calculated by subtracting the amount of time spent in MVPA according to the GPAQ from the amount spent in MVPA according to accelerometry. Variables considered as potential predictors included age, education, BMI, and whether their partner lived in the household, round, topography, and food security. The median village altitude was used as an indicator of topography. Food security was measured using the food gap; a subjective indicator used widely in Ethiopia [40]. Backward-stepwise regression was used to build the model; variables were retained in the model if their *p* value was <0.05. Prediction models were considered for the 2 rounds combined and each round individually. Interaction terms for the round were considered in the combined model.

### Twenty-four-hour PERS analysis

Reliability was not calculated for PERS because each response corresponded to a different 24-h period. The validity of the PERS was determined by comparing the proportion of time participants reported spending at each level of exertion to the proportion of time recorded by accelerometry. The accelerometry data were restricted to the same time frame as the recall. Values were compared using both Pearson’s correlation coefficients and Spearman’s rank correlation coefficients. For the PERS to be valid, the correlation coefficient needed to be moderate/acceptable.

Multivariable regression modeling was used to create a calibrated recall value. The observations were split into training (80%) and testing (20%) samples. The training sample was used to create the regression model to calibrate the recall data with the results of accelerometry as the dependent variable and the results of the recall data and participant characteristics as independent variables. The model was fit using backward-stepwise regression that used the Bayesian information criterion to select the best-fit model. Random effect and fixed effect models were considered to address potential independence issues resulting from participants who appeared twice in the dataset during the dry season round and again during the agricultural round.

This study received ethical approval from the IRB at Cornell University (1902008596) and the International Food Policy Research Institute (DSG-19-0203P).

## Results

### Study population

Demographic characteristics of the study population appear in Table 1. Accelerometry was collected on 95 women during the dry season round and 96 during the agricultural round. There were valid accelerometry data from 89 women from the dry season round and 91 from the agricultural round. Sixty-four women participated in both rounds. Twenty-five women only participated in the dry season round, and 27 women only participated in the agricultural round.

**TABLE 1:** Summary of study participants’ characteristics

Characteristic	Dry season round	Agricultural round
*n*	89	91
Age	29 (7)	30 (7)
Any education	47.8%	44.4%
Partner lives in the household	87.8%	85.5%
BMI (kg/m^2^)	19.4 (2.1)	19.4 (2.0)
Underweight (BMI < 18.5 kg/m^2^)	33.3%	33.0%
Minimum dietary diversity	4.4%	4.4%
Lactating	97.9%	89.4%
Household size	5.5 (1.8)	5.7 (1.7)
Male-headed households	90.0%	85.6%
Households were smallholder farmers	84.4%	83.3%
Benefits from public works safety net	48.3%	45.5%
Food insecure during the past 6 mo	32.2%	28.9%
Food groups consumed	3.0 (0.5)	3.2 (0.8)
Distance to the nearest city with 50,000 inhabitants (km)	67.3 (28.0)	66.5 (27.9)
Travel time to market (min)	44.9 (34.5)	44.6 (34.8)
Time to fetch water and return (min)	44.0 (39.0)	42.8 (31.4)
Houses with no or open windows	62.2%	61.1%
Houses with dirt floors	67.8%	67.8%
Houses with corrugated metal roofs	53.3	61.1%
Access to electricity	43.3%	44.4%
Owned mobile phone	67.8%	66.7%

Values are presented as means with SD in parentheses or as a percent of the study population.

The average age of the study participants was 30 y. The average BMI was 19.4 kg/m^2^, and ∼33% of women were underweight (BMI < 18.5 kg/m^2^). Dietary diversity was measured using the Minimum Dietary Diversity-Women [54]. Overall, dietary diversity was low; only 4.4% of women met the minimum dietary diversity by consuming food from ≥5 of the 10 recommended food groups. The change in lactation status between the dry season, 97.8%, and the agricultural season, 89.4%, may be due to the increasing age of their children. Typical of the region, 83.9% of households were actively engaged in farming. Less than half, 46.7%, were participants in the public works component of the PSNP.

### GPAQ reliability

The GPAQ had moderate/acceptable or better agreement for almost all dichotomous responses (Table 2). The agreement was only fair for travel during the dry season round (κ = 0.33). There was a poor agreement for vigorous recreation and a poor-to-fair agreement for moderate recreation. Less than 2% of participants said that they engaged in moderate or vigorous recreational activities. Except for the time spent in moderate or vigorous levels of exertion during recreation, the number of minutes spent in moderate or vigorous exertion for work, travel, and in total had at least moderate/acceptable agreement (Table 3). During the agricultural round, there was a near-perfect agreement (Spearman’s rank ρ = 0.81) for the number of minutes respondents reported spending at moderate exertion work.

**TABLE 2:** Reliability of the GPAQ for dichotomous responses

Domain	Exertion level	All	Dry season round	Agricultural round
*n*		185	93	92
Work				
	Vigorous	0.63∗∗∗ (85.41%)	0.57∗∗∗ (81.72%)	0.69∗∗∗ (89.13%)
	Moderate	0.63∗∗∗ (83.78%)	0.46∗∗∗ (76.34%)	0.80∗∗∗ (91.30%)
Travel		0.69∗∗∗ (87.03%)	0.33∗∗∗ (82.80%)	0.82∗∗∗ (91.30%)
Recreation^1^				
	Vigorous	−0.02 (95.68%)	−0.03 (94.62%)	−0.01 (96.74%)
	Moderate	0.39∗∗∗ (98.38%)	0.49∗∗∗ (97.85%)	0.00 (98.91%)

GPAQ, global physical activity questionnaire. The κ for each response is shown with the percent of agreement below. ^1^Less than 5 participants said that they participated in vigorous or moderate recreation during each visit of each round. ∗∗∗ Indicates statistical significance <0.01.

**TABLE 3:** Reliability of the GPAQ for continuous responses—Spearman’s rank ρ

Domain	Exertion level	All	Dry season round	Agricultural round
*n*		185	93	92
Work				
	Vigorous	0.64∗∗∗	0.45∗∗	0.78∗∗∗
	Moderate	0.74∗∗∗	0.64∗∗∗	0.81∗∗∗∗
Travel		0.69∗∗∗	0.50∗∗	0.84∗∗∗∗
Recreation^1^				
	Vigorous	−0.02	−0.03	−0.02
	Moderate	−0.02	−0.03	−0.02
Total minutes				
	Vigorous	0.61∗∗∗	0.44∗∗	0.72∗∗∗
	Moderate	0.69∗∗∗	0.60∗∗	0.75∗∗∗
	Sedentary/light	0.67∗∗∗	0.69∗∗∗	0.62∗∗∗

GPAQ, global physical activity questionnaire. ∗ Indicates fair agreement, ∗∗ indicates moderate/acceptable agreement, ∗∗∗ indicates substantial agreement, and ∗∗∗∗ indicates near-perfect agreement.

1 Less than five participants said they participated in vigorous or moderate recreation during each visit of each round.

### GPAQ validity

There was a lack of agreement between the proportion of minutes spent at each level of exertion according to the GPAQ and accelerometry (Table 4). When the 2 rounds were combined, the average proportion of time spent at sedentary/light and moderate exertion was similar when measured by the GPAQ (88.9%) and accelerometry (90.2%). Despite the similarities in the average proportion of time spent at each exertion level, there was poor agreement. Pearson’s correlation coefficient never exceeded 0.20, and Spearman’s rank correlation coefficient never exceeded 0.18.

**TABLE 4:** Validity of GPAQ

Exertion level	GPAQ	Accelerometry	Pearson’s ρ	Spearman’s rank ρ
Total				
*n*	180	180		
Proportion of time at sedentary/light exertion	88.9%	90.2%	−0.06	−0.02
Proportion of time at moderate exertion	9.5%	9.3%	−0.12	−0.06
Proportion of time at vigorous exertion	1.6%	0.4%	0.06	0.16
Proportion of time at MVPA	11.1%	9.8%	−0.06	−0.02
Dry season round				
*n*	89	89		
Proportion of time at sedentary/light exertion	91.8%	90.6%	−0.11	0.10
Proportion of time at moderate exertion	6.7%	9.6%	−0.01	0.05
Proportion of time at vigorous exertion	1.4%	0.4%	0.20	0.18
Proportion of time at MVPA	8.2%	10.0%	0.07	0.10
Agricultural round				
*n*	91	91		
Proportion of time at sedentary/light exertion	86.0%	90.4%	−0.11	−0.09
Proportion of time at moderate exertion	12.1%	9.0%	−0.15	−0.13
Proportion of time at vigorous exertion	1.8%	0.5%	0.01	0.15
Proportion of time at MVPA	14.0%	9.6%	−0.11	−0.09

GPAQ, global physical activity questionnaire; MVPA, moderate or vigorous physical activity. ∗ Indicates fair agreement, ∗∗ indicates moderate/acceptable agreement, ∗∗∗ indicates substantial agreement, and ∗∗∗∗ indicates near-perfect agreement.

### GPAQ discordancy

During the dry season round, on average, participants underestimated the proportion of time spent at MVPA by 2%. During the agricultural round, on average, participants overestimated the proportion of time spent at MVPA by 4%. In all 3 models, each round separately and the rounds combined, the proportion of time spent at MVPA according to the accelerometer was a significant predictor of discordancy in a multivariate model (Table 5). When both rounds were combined, the round was also a significant predictor of discordancy. The discordancy between rounds was evaluated using Spearman’s rank correlation coefficient. Spearman’s rank correlation coefficient was 0.38, which is a fair agreement.

**TABLE 5:** Predictors of discordancy in GPAQ

Model parameter	Univariate model			Multivariate model		
	Coefficient	95% CI	*P* value	Coefficient	95% CI	*P* value
Round	−0.06	(−0.10, 0.03)	<0.01∗∗∗	−0.06	(−0.09, −0.03)	<0.01∗∗∗
Proportion of time at MVPA—accelerometry	1.19	(0.79, 1.59)	<0.01	1.15	(0.76, 1.53)	<0.01∗∗∗
BMI	−0.01	(−0.02, −0.00)	0.02∗∗			
Partner in household	0.06	(0.01, 0.10)	0.02∗∗			
Age	−0.00	(−0.00, 0.00)	0.81			
Education	0.00	(−0.00, 0.01)	0.64			
Altitude	0.00	(−0.00, 0.00)	0.24			
Food insecurity	0.02	(0.00, 0.03)	0.03∗∗			
Public works	0.01	(−0.02, 0.05)	0.52			
Dry season round						
Proportion of time at MVPA—accelerometry	0.86	(0.46, 1.26)	<0.01∗∗∗	0.86	(0.46, 1.26)	<0.01∗∗∗
BMI	−0.00	(−0.01, 0.00)	0.39			
Partner in household	0.05	(0.00, 0.10)	0.03∗∗			
Age	0.00	(−0.00, 0.00)	0.30			
Education	0.00	(−0.00, 0.00)	0.83		—	—
Altitude	0.00	(−0.00, 0.00)	0.16∗			
Food insecurity	0.00	(−0.01, 0.02)	0.49			
Public works	−0.01	(−0.04, 0.02)	0.55			
Agricultural round						
Proportion of time at MVPA—accelerometry	1.36	(0.72, 1.99)	<0.01∗∗∗	1.36	(0.72, 1.99)	<0.01∗∗∗
BMI	−0.02	(−0.03, −0.00)	0.02∗∗			
Partner in household	0.06	(−0.02, 0.14)	0.15∗			
Age	−0.00	(−0.01, 0.00)	0.55			
Education	0.00	(−0.00, 0.01)	0.42			
Altitude	0.00	(−0.00, 0.00)	0.49			
Food insecurity	0.03	(0.00, 0.06)	0.03∗∗			
Public works	0.03	(−0.03, 0.09)	0.35			

GPAQ, global physical activity questionnaire; MVPA, moderate or vigorous physical activity. ∗ Indicates statistical significance <0.20, qualifying for consideration in the multivariate model, ∗∗ indicates statistical significance at <0.05, and ∗∗∗ indicates statistical significance at <0.01.

### Twenty-four-hour PERS validity

Participants overestimated the proportion of time spent at moderate or vigorous exertion and underestimated the proportion of time spent at sedentary/light exertion (Table 6). When both rounds were combined, participants recalled spending on an average 23%, whereas accelerometry measured 13% of their time in MVPA. There was fair agreement between these values (Spearman’s rank ρ = 0.25).

**TABLE 6:** Validity of 24-h recall

Exertion level	24-h recall	Accelerometry	Pearson’s ρ	Spearman’s rank ρ
Total				
*n*	180	180		
Proportion of time at sedentary/light exertion	77%	87%	0.26∗	0.25∗
Proportion of time at moderate exertion	20%	12%	0.19	0.20
Proportion of time at vigorous exertion	3%	1%	0.15	0.24∗
Proportion of time at MVPA	23%	13%	0.26∗	0.25∗
Dry season round				
*n*	89	89		
Proportion of time at sedentary/light exertion	83%	89%	0.25∗	0.26∗
Proportion of time at moderate exertion	16%	11%	0.20	0.23∗
Proportion of time at vigorous exertion	1%	0%	0.01	0.18
Proportion of time at MVPA	17%	11%	0.25∗	0.27∗
Agricultural round				
*n*	91	91		
Proportion of time at sedentary/light exertion	72%	85%	0.19	0.16
Proportion of time at moderate exertion	23%	14%	0.13	0.10
Proportion of time at vigorous exertion	5%	1%	0.09	0.22∗
Proportion of time at MVPA	28%	15%	0.19	0.16

MVPA, moderate or vigorous physical activity. ∗ Indicates fair agreement, ∗∗ indicates moderate/acceptable agreement, ∗∗∗ indicates substantial agreement, and ∗∗∗∗ indicated near-perfect agreement.

Participants spent more time in MVPA during the agricultural round than during the dry season round, 15% compared with 11% according to accelerometry, and 28% compared with 17% according to the 24-h recall. During the dry season round, there was a fair agreement between the reported and the measured proportion of the time at MVPA (Spearman’s rank ρ = 0.27). During the agricultural round, there was poor agreement between the participants’ self-reported proportion of time spent at MVPA compared with accelerometry (Spearman’s rank ρ = 0.16).

### Twenty-four-hour PERS calibration

**FIGURE 1:**
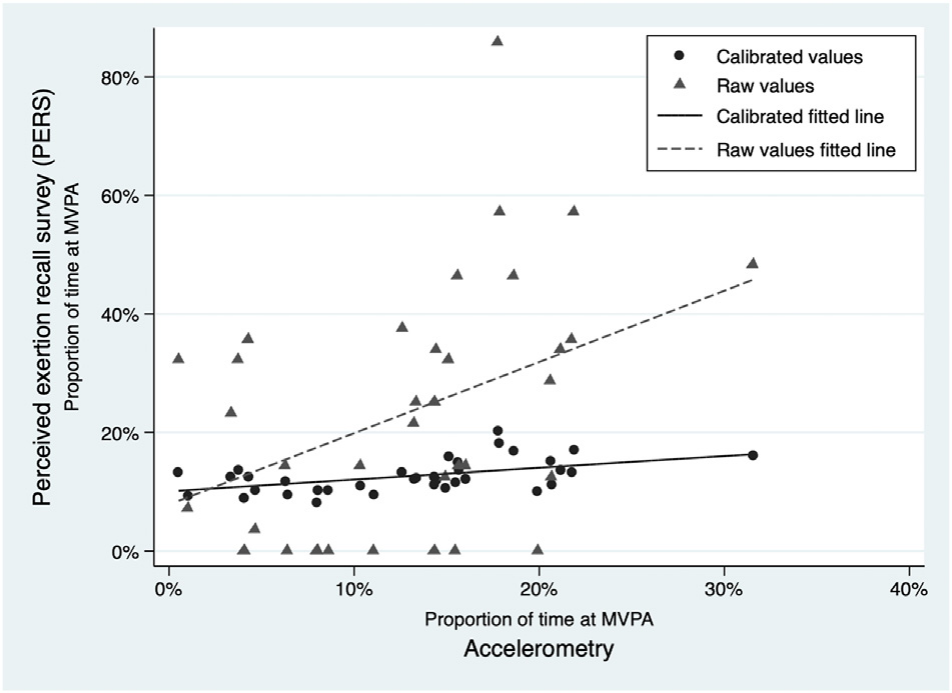
Proportion of time spent at MVPA recorded by accelerometry and the 24-h PERS. MVPA, moderate or vigorous physical activity; PERS, perceived exertion recall survey.

BMI was the only significant predictor of MVPA according to accelerometry when fit into a linear model with the proportion for time spent at MVPA according to the 24-h recall of exertion (Table 7). Breakpoint analysis indicated a breakpoint of 0.00 and was not used. Random effects had no impact on the coefficients or *p* values of the covariates in the model. When applied to the testing set of participants, the calibration of the model using BMI increased Spearman’s rank ρ to 0.53, increasing the agreement to moderate/acceptable. The increase in the correlation between the raw and calibrated values is evident when the values are plotted (Figure 1).

**TABLE 7:** Calibration of the 24-h recall model

Model parameter	Univariate model			Multivariate model		
	Coefficient	95% CI	*P* value	Coefficient	95% CI	*P* value
*n* = 144						
Proportion MVPA according to 24-h recall∗	0.12	(0.08, 0.16)	<0.01∗∗∗	0.12	(0.08, 0.17)	<0.01∗∗∗
BMI	−0.00	(−0.01, −0.00)	0.08∗∗	−0.01	(−0.01, −0.00)	0.01∗∗
Round	0.04	(0.02, 0.06)	<0.01∗∗∗			
Partner in household	0.01	(−0.02, 0.05)	0.44			
Age	−0.00	(−0.00, 0.00)	0.18∗			
Education	−0.01	(−0.03, 0.02)	0.58			
Altitude	−0.00	(−0.00, 0.00)	0.43			
Food insecurity	0.00	(−0.01, 0.01)	0.69			
Public works	−0.01	(−0.03, 0.01)	0.40			

MVPA, moderate or vigorous physical activity. ∗ Indicates statistical significance <0.20, qualifying for consideration in the multivariate model, ∗∗ indicates statistical significance at <0.05, and ∗∗∗ indicates statistical significance at <0.01.

## Discussion

The regular activities of women’s lives in rural highland Ethiopia are physically demanding, complicating efforts to improve their health and nutritional status. Although increased energy and micronutrients are important for health and economic productivity [2], it is challenging to identify those with greater energy needs without insight into their physical activity. Therefore, this study aimed to use accelerometers to validate 2 survey tools, the WHO’s GPAQ and the 24-h PERS. The GPAQ captures the average time spent in each level of exertion in a typical day during a typical week. The PERS collects recall data from the previous 24 h in 30-min epochs and includes time use and activity prompts.

### Findings

The GPAQ was reliable but lacked validity when compared with accelerometry. In this study population, the GPAQ was not a valid method for measuring exertion. Discordancy analysis showed that participants who spent a greater proportion of their time at MVPA tended to underestimate it and vice versa.

The calibrated 24-h PERS was a valid tool to measure the proportion of time spent in MVPA in women living in rural highland Ethiopia. When adjusted for BMI, the survey identified members of the study population who spent an increased proportion of their time at higher levels of exertion.

The rationale for using a 24-h recall for perceived exertion is similar to those used for a 24-h recall of dietary intake [55]. A single 24-h recall per participant can be used to characterize the average of a group, provided the sample represents the underlying population of interest and variations throughout the week and timeframe of interest [[56], [57], [58], [59]].

### Future directions

A previous multicountry study found that the GPAQ was invalid in many settings [22]. This study improved upon the work in Ethiopia by using triaxial accelerometers rather than pedometers, though with similar results. The GPAQ is predominantly used to identify sedentary populations [28,60] or assess the effectiveness of interventions promoting physical activity [25]. In contrast, the 24-h PERS identifies people spending a larger proportion of their time at MVPA. Identifying individuals whose life circumstances or livelihoods are energy intensive will assist in identifying potential barriers to success for interventions that aim to improve nutrition and health outcomes. Future research should seek to replicate the calibration of the 24-h PERS for use in other contexts.

The discordancy analysis of the GPAQ found seasonal variations in the relationship between actual and perceived exertion that should be further examined. Participants overestimated the proportion of their time spent at MVPA more in the agricultural round when they were more likely to be engaged in food production activities than childcare or personal care activities [40]. Additional research is needed to determine if participants over- or underestimated their time spent in MVPA because of a training effect [61,62], the type of activities they were engaged in, or an underlying seasonal effect such as food security, caloric intake [63], or diet composition [64].

The significance of the BMI-calibrated model of the 24-h PERS demonstrates that as BMI increased, so did the difference in the proportion of time spent at MVPA recalled by the participants and recorded by accelerometry. This could be due to the positive relationship between activity-induced energy expenditure and body mass [65]. A meta-analysis concluded that there was a valid relationship between actual and perceived exertion in overweight and obese individuals [66]. Future research should be conducted to understand the relationship between actual exertion and perceived exertion across a wide range of BMI in various settings.

### Strengths and limitations

This study improves upon previous efforts to validate the GPAQ in Ethiopia by using triaxial accelerometers rather than pedometers [22]. In addition, GPAQ discordancy analysis identified participant characteristics that contribute to the lack of validity, allowing for potential future iterations of the GPAQ or similar surveys to incorporate them.

This study piloted a novel survey module that can be used to estimate the proportion of time spent at MVPA. The 24-h PERS had many characteristics that likely contributed to its validity that the GPAQ lacked, including a shorter recall time, prompts that aided recall, and more detailed questions regarding their energy exertion. In addition, the 24-h PERS only required the participants to recall their activity from the previous day instead of asking about an average week. To our knowledge, this is the first study to attempt to calibrate participant-reported physical activity data using participant characteristics. The improvement in the agreement between the raw and calibrated values demonstrates this method’s potential to improve the validity of the data collected.

Ethiopia is a large and diverse country; this study took only 1 region, Tigray. The lack of heart rate data is 1 limitation of this survey that lessens the ability of the accelerometers to calculate METs and may result in misclassification bias. Similar to a 24-h recall of dietary intake, a 24-h PERS may be open to systematic bias and random error [[67], [68], [69], [70], [71]]. In addition, although the sample size was adequate, additional participants might have allowed more precise estimates of associations. Another limitation is the lack of data on breastfeeding frequency [72]. Future research should include breastfeeding frequency and other participant characteristics, including body composition [73], physical function [62], anemia [74], and overall health.

The sample had some different participants in each round despite efforts to use the same participants (Supplemental Table 1). There were no differences in the participant characteristics between the samples for each round. A sensitivity analysis disaggregated the data based on which rounds the participants were in. The findings for the GPAQ remained the same (Supplemental Table 2). The 24-h PERS had a lower agreement when disaggregated by rounds and by those that appeared in both rounds compared with only 1 (Supplemental Table 3).

Most importantly, the results of the 24-h PERS to determine the exact levels of physical activity or energy expenditure should not be overstated. The agreement is sufficient to compare the time spent at MVPA between groups and seasons in women living in the rural highland regions of Ethiopia, but caution should be used for any further analysis.

In conclusion, this study validated a new survey tool, the 24-h PERS, to estimate the proportion of time spent at moderate and vigorous physical activity in women living in rural highland Ethiopia. This tool can be used in future research to understand the demands of life in the region. The survey will allow comparisons between groups of people to determine if there is a difference in the physical activity of women participating in manual labor, such as that required by the PSNP. Understanding physical activity levels is critical to addressing undernutrition in this region and will allow researchers to look beyond consumption patterns and into expenditure.

## Author disclosures

The authors report no conflicts of interest.

## Data Availability

Data described in the manuscript, code book, and analytic code will be available upon request pending application and approval.

## Appendix A Supplementary data

Multimedia component1 Multimedia component1
