# Supplementary material for: Validation of the 24-h perceived exertion recall survey in women in rural Tigray, Ethiopia
Source: Curr Dev Nutr. 2023 Mar 8;7(7):100064. doi: 10.1016/j.cdnut.2023.100064 (PMC10401292; doi:10.1016/j.cdnut.2023.100064)
Supplement: Multimedia component1 [file mmc1.docx]

Supplemental material for online publication

| Table S1: Study population characteristics by round in which participants appear | | | |
| --- | --- | --- | --- |
| Characteristic | Both round | Dry Season Round only | Agricultural Round only |
| **N** | **64** | **25** | **27** |
| Age | 30 | 29 | 31 |
| Any education | 47.7 % | 48.0% | 37.0% |
| BMI (^kg^/_m_^2^) | 19.5 | 19.2 | 19.3 |
| Underweight (BMI < 18.5 ^kg^/_m_^2^) | 26.6 % | 44.0 % | 29.6% |
| Partner lives in household | 87.5 % | 88.0 % | 81.5 % |
| Food insecure during past 6 months | 31.2 % | 28.0 % | 26.9 % |
| Benefits from public works safety net | 46.9 % | 44.0 % | 48.1% |
| Breastfed during the dry season | 98.6% | 95.5% |  |
| Breastfed during the agricultural season | 91.8% |  | 81.0% |

| Table S2: GPAQ validity disaggregated by round in which participants participated | | | | | |
| --- | --- | --- | --- | --- | --- |
| Exertion level | GPAQ | Accelerometry | Pearson’s ρ | Spearman’s rank ρ |  |
| Dry Season Round |  |  |  |  |  |
| Both rounds |  |  |  |  |  |
| **N** | **64** | **64** |  |  |  |
| Proportion of time at sedentary/light exertion | 92.9 | 90.0 | 0.07 | 0.12 |  |
| Proportion of time at moderate exertion | 5.7 % | 9.6 % | 0.02 | 0.10 |  |
| Proportion of time at vigorous exertion | 1.4 % | 0.4 % | 0.08 | 0.12 |  |
| Proportion of time at MVPA | 7.1 % | 10.0 % | 0.07 | 0.12 |  |
| Dry Season Round only |  |  |  |  |  |
| **N** | **25** | **25** |  |  |  |
| Proportion of time at sedentary/light exertion | 89.3 | 90.0 | 0.08 | 0.05 |  |
| Proportion of time at moderate exertion | 8.9 % | 9.6 % | -0.09 | -0.06 |  |
| Proportion of time at vigorous exertion | 1.7 % | 0.4 % | 0.49 | 0.37 |  |
| Proportion of time at MVPA | 10.7 % | 10.0 % | 0.08 | 0.05 |  |
| Agricultural Round |  |  |  |  |  |
| Both rounds |  |  |  |  |  |
| **N** | **64** | **64** |  |  |  |
| Proportion of time at sedentary/light exertion | 87.1 | 91.0 | -0.15 | -0.14 |  |
| Proportion of time at moderate exertion | 11.7 % | 8.5 % | -0.14 | -0.16 |  |
| Proportion of time at vigorous exertion | 1.2 % | 0.5 % | -0.14 | 0.01 |  |
| Proportion of time at MVPA | 12.9 % | 10.0 % | -0.15 | -0.14 |  |
| Agricultural Round only |  |  |  |  |  |
| **N** | **27** | **27** |  |  |  |
| Proportion of time at sedentary/light exertion | 83.7 | 89.0 | -0.14 | -0.10 |  |
| Proportion of time at moderate exertion | 13.1 % | 10.4 % | -0.29 | -0.20 |  |
| Proportion of time at vigorous exertion | 3.2 % | 0.6 % | 0.19 | 0.25 |  |
| Proportion of time at MVPA | 16.3 % | 11.0 % | -0.14 | -0.10 |  |

| Table S3: 24-hour recall validity disaggregated by round in which participants participated | | | | |
| --- | --- | --- | --- | --- |
| Exertion level | 24-hour recall | Accelerometry | Pearson’s ρ | Spearman’s rank ρ |
| Dry Season Round |  |  |  |  |
| Both rounds |  |  |  |  |
| **N** | **64** | **64** |  |  |
| Proportion of time at sedentary/light exertion | 86.2 % | 89.1 % | 0.34 | 0.32 |
| Proportion of time at moderate exertion | 13.2 % | 10.5 % | 0.27 | 0.27 |
| Proportion of time at vigorous exertion | 0.6 % | 0.4 % | 0.02 | 0.20 |
| Proportion of time at MVPA | 13.8 % | 10.9 % | 0.34 | 0.32 |
| Dry Season Round only |  |  |  |  |
| **N** | **25** | **25** |  |  |
| Proportion of time at sedentary/light exertion | 76.3 % | 88.8 % | 0.06 | 0.07 |
| Proportion of time at moderate exertion | 23.4 % | 10.9 % | 0.06 | 0.07 |
| Proportion of time at vigorous exertion | 0.3 % | 0.4 % | -0.07 | 0.12 |
| Proportion of time at MVPA | 23.7 % | 11.2 % | 0.06 | 0.07 |
| Agricultural Round |  |  |  |  |
| Both rounds |  |  |  |  |
| **N** | **64** | **64** |  |  |
| Proportion of time at sedentary/light exertion | 75.4 % | 85.9 % | 0.13 | 0.15 |
| Proportion of time at moderate exertion | 20.6 % | 13.3 % | 0.09 | 0.09 |
| Proportion of time at vigorous exertion | 4.0 % | 0.8 % | 0.06 | 0.21 |
| Proportion of time at MVPA | 24.6 % | 14.1 % | 0.13 | 0.15 |
| Agricultural Round only |  |  |  |  |
| **N** | **27** | **27** |  |  |
| Proportion of time at sedentary/light exertion | 62.8 % | 83.1 % | 0.25 | 0.18 |
| Proportion of time at moderate exertion | 29.3 % | 16.1 % | 0.12 | 0.03 |
| Proportion of time at vigorous exertion | 7.9 % | 0.9 % | 0.15 | 0.20 |
| Proportion of time at MVPA | 37.2 % | 16.9 % | 0.25 | 0.17 |
